# Prognostic significance of systemic inflammation response index, systemic immune-inflammation index and neutrophil-to-lymphocyte ratio in patients with chronic subdural hematoma after burr hole drainage

**DOI:** 10.3389/fneur.2025.1740766

**Published:** 2026-01-12

**Authors:** Yong Gu, Xiaojiang Yu, Xiaodong Long

**Affiliations:** 1 Department of Neurosurgery, People’s Hospital of Deyang City, Deyang, China; 2 Information of Engineering, People’s Hospital of Deyang City, Deyang, China

**Keywords:** chronic subdural hematoma, neutrophil-to-lymphocyte ratio, poor prognosis, systemic immune-inflammation index, systemic inflammation response index

## Abstract

**Background and purpose:**

Systemic Inflammation Response Index (SIRI), Systemic Immune-Inflammation Index (SII), and Neutrophil-to-Lymphocyte Ratio (NLR) are novel immune inflammatory markers that have been proven to have excellent predictive value for many diseases. The aim of this study was to investigate the prognostic value of SIRI, SII, and NLR for functional outcome at 1 month post-discharge in chronic subdural hematoma (CSDH) patients after burr hole draining.

**Methods:**

This retrospective analysis used a database of CSHD patients who underwent burr hole drainage from the Department of Neurosurgery at Deyang People’s Hospital from June 1, 2019 to December 31, 2023. Poor prognosis was defined as a Modified Rankine Scale (mRS) score of 3–6 at 1 month post-discharge. Univariate analysis and multivariate logistic regression analysis were used to assess the correlation between the indicators and outcomes. Harrell’s concordance index (C-index), and Akaike information criterion (AIC) were used to assess the predictive accuracy and model-fitting of predictive models.

**Results:**

A total of 445 patients were enrolled in the final analysis, of whom 90 (20.22%) developed poor prognosis. SIRI (*p* = 0.019), and NLR (*p* = 0.045) were independent risk factors for poor prognosis. The Area Under the Curve (AUC) value of SIRI was 0.765 and the optimal cutoff point was 3.09 × 109/L. The AUC value of NLR was 0.771 and the optimal cutoff point was 5.53. The “Basic model + NLR” had the C-index value (0.861) and AIC value (325.61). The “Basic model + SIRI” had the C-index value (0.862) and AIC (321.98). The “Basic model + SIRI + NLR” had the C-index value (0.865) and AIC value (322.00).

**Conclusion:**

To our knowledge, this is the first study to examine the prognostic significance of admission SIRI in CSDH patients. In this study, SIRI and NLR had prognostic significance in CSDH patients after burr hole drainage. When combined with the basic model (history of ischemic stroke, brain herniation, admission GCS, anticoagulant or antiplatelet therapy), the SIRI has better predictive accuracy (C-index 0.862 vs. 0.861) and model-fitting (AIC 321.98 vs. 325.61) than NLR.

## Introduction

Chronic subdural hematoma (CSDH) is the accumulation of blood and degraded blood products in the subdural space (1, 2). Previous research has shown that the average yearly incidence of CSDH ranges between 1.7 and 20.6 per 100,000 people (3). Older persons (>65 years) are more sensitive to CSDH due to increasing anticoagulant therapy, brain atrophy, and an increased risk of head trauma from falls (4). Common symptoms of CSDH include focal neurological impairments, changed mental status, and symptoms linked with high intracranial pressure, such as headache, decreased consciousness and brain function, and even death (2, 5). In the past 20 to 30 years, the incidence of CSDH has almost tripled due to aging, and the number of surgeries has doubled (2, 6). These findings are consistent with the rising frequency of CSDH in the United States, where the number of cases is anticipated to reach 60,000 year by 2030 (7).

Although some medications (such as atorvastatin calcium and dexamethasone) are used to treat CSDH, surgery is still the first option for patients with symptomatic CSDH (8, 9). Burr-hole drainage is the most used surgical technique in clinical practice (5). However, some patients have a poor prognosis and postoperative recurrence, despite the fact that the great majority of patients have favorable surgical results. The literature reports relapse rates ranging from 5 to 33% (10, 11). Patients with CSDH who have a poor prognosis also have a higher risk of dying (12, 13). Previous studies have found that age, Glasgow Coma Scale (GCS) score at admission, midline shift, and history of alcohol abuse are significantly associated with CSDH outcomes (13–16). Therefore, improving the prognosis of CSDH patients following surgery is a critical issue in treatment.

Growing evidence has indicated that immune and inflammatory responses participate in the pathophysiological processes of CSDH, and inflammation and immunity are crucial contributors to postoperative recurrence and poor prognosis (17–20). Studies have shown that the membranes of CSDH are infiltrated by inflammatory and immune cells, such as neutrophils, lymphocytes, macrophages, eosinophils, etc. (21, 22). The inflammatory process involved in CSDH membrane and fluid formation is localized to the subdural space (19). As previously stated, inflammation in CSDH is caused by a range of inflammatory and immunological cells, including neutrophils, lymphocytes, macrophages, and eosinophils (20, 23, 24). These white blood cells deposit and release a large number of cytokines, including chemokines, which contribute to the aggregation of inflammatory and immunological cells (18). Analysis of pro-inflammatory and anti-inflammatory cytokines in CSDH revealed that both were much greater than those in peripheral blood, although pro-inflammatory cytokines were significantly higher than anti-inflammatory cytokines (18). The balance of this inflammatory and immunological response, as well as its evolution over time, have a substantial impact on the prognosis of CSDH (18, 20, 25). Previous research has demonstrated that cerebral local inflammation is associated with systemic inflammatory responses as evaluated by changes in white blood cells and vital signs, which can assist in predicting patient outcomes (26, 27).

The Systemic Inflammatory Response Index (SIRI) is a novel systemic inflammatory marker based on peripheral blood neutrophil, monocyte, and lymphocyte counts (calculated as neutrophil count × monocyte count/lymphocyte count) (28, 29). The systemic immune-inflammation index (SII), which is calculated as platelet count × monocyte count/lymphocyte count, is a novel immune-inflammatory marker that can more comprehensively reflect the balance between immune and inflammatory responses (30). The neutrophil-to-lymphocyte ratio (NLR) is considered an important parameter for assessing systemic inflammatory status and infection risk (31). In previous studies, SIRI, SII, and NLR were discovered to be independent prognostic indications for a wide range of disorders, including glioma, pancreatic cancer, cerebral hemorrhage, ruptured intracranial aneurysms, acute ischemic stroke, pulmonary embolism, and others (32–37).

However, few studies have been reported on the prognostic significance of SIRI, SII, and NLR in patients with CSDH after burr hole drainage. Therefore, the purpose of this study was to evaluate the prognostic significance of admission SIRI, SII, and NLR in patients with CSDH after burr hole drainage.

## Materials and methods

### Study design

This retrospective analysis used a prospective database of CSHD patients who underwent burr hole drainage from the Department of Neurosurgery at Deyang People’s Hospital from June 1, 2019, to December 31, 2023. The baseline clinical data were obtained from the electronic medical record system at Deyang People’s Hospital.

The exclusion criteria are listed below: (1) Bilateral chronic subdural hematoma; (2) Incomplete baseline clinical data; (3) Coexisting with tumors, aneurysms, arteriovenous malformations, and other diseases; (4) Lack of blood test results and head CT follow-up within 24 h of admission; (5) History of infectious diseases, cancer, rheumatic diseases, blood system diseases, or other diseases that significantly affect peripheral blood cells; (6) Loss of follow-up.

### Clinical parameter assessment

Clinical variables were obtained from the electronic medical record system, including the following variables: (1) demography: age at onset, gender, height, weight, and BMI; (2) clinical history: hypertension, diabetes, smoking, drinking, cerebral infarction, anticoagulant and antiplatelet medication use. (3) admission conditions: Glasgow Coma Scale (GCS) score; (4) imaging characteristics of cerebral hemorrhage: hematoma thickness, presence of cerebral hernia; (5) treatment status; (6) routine blood tests: it is vital to note that standard blood tests are conducted within 8 h following admission. SIRI was defined as neutrophil count × monocyte count/lymphocyte count. SII is calculated as platelet count × monocyte count/lymphocyte count. NLR is the neutrophil-to-lymphocyte ratio.

Our center routinely uses a hand drill for single-hole drainage of chronic subdural hematoma. We rinse with normal saline until clear liquid is discharged. After the operation, a subdural closed drainage was performed. The duration of the drainage was determined based on the results of the postoperative head CT examination.

The patients were followed up once a month after discharge, and the primary outcome was functional status 1 month later. Patients’ functional outcomes were assessed at each follow-up using the modified Rankine Scale (mRS). A good prognosis was defined as an mRs score of 0–2, and a poor prognosis as an mRS score of 3–6 (38).

### Statistical analysis

All statistical analyses were carried out using SPSS software (version 22.0; IBM, Armonk, NY, USA) and R software (version 3.6.1). Continuous variables are displayed as mean ± SD or median with interquartile range (IQR), while categorical variables are represented as frequency and percentage. Categorical variables were compared with the χ2 or Fisher’s exact test. Continuous variables that followed the normal distribution were compared using the Student’s *t*-test; otherwise, the Mann–Whitney *U*-test was used. Logistic regression analyses were conducted to examine the impact of risk factors on CSDH outcomes. Variables with *p* < 0.05 in univariate analysis were included in the backward stepwise multivariate logistic regression. A receiver operating characteristic (ROC) curve was performed to determine the accuracy of the SIRI and NLR. The optimal cut-off values of SIRI and NLR were determined by calculating the maximum Youden index using the ROC curve. Predictive models for outcomes were composed of independent predictive indicators in multivariate logistic regression. The basic model included 4 independent variables (history of ischemic stroke, brain herniation, admission GCS, and anticoagulant or antiplatelet therapy) but exclude peripheral blood indicators. Harrell’s concordance index (C-index) and Akaike information criterion (AIC) were used to assess the predictive accuracy and model fitting of predictive models, respectively. Higher C-index indicated better predictive accuracy, and lower AICs indicated superior model-fitting (39, 40). A two-sided *p* < 0.05 was considered statistically significant. Based on the multivariate regression analysis results, a nomogram for poor prognosis probability was constructed.

### Ethics

This study protocol was approved by the Institutional Ethics Committee of Deyang People’s Hospital (No. 2024–04-116-K01) and conducted in accordance with the principles outlined in the Declaration of Helsinki. All medical record data were anonymized to protect patient privacy, thereby waiving the requirement for informed consent.

## Results

### Baseline characteristics

**Figure 1:**
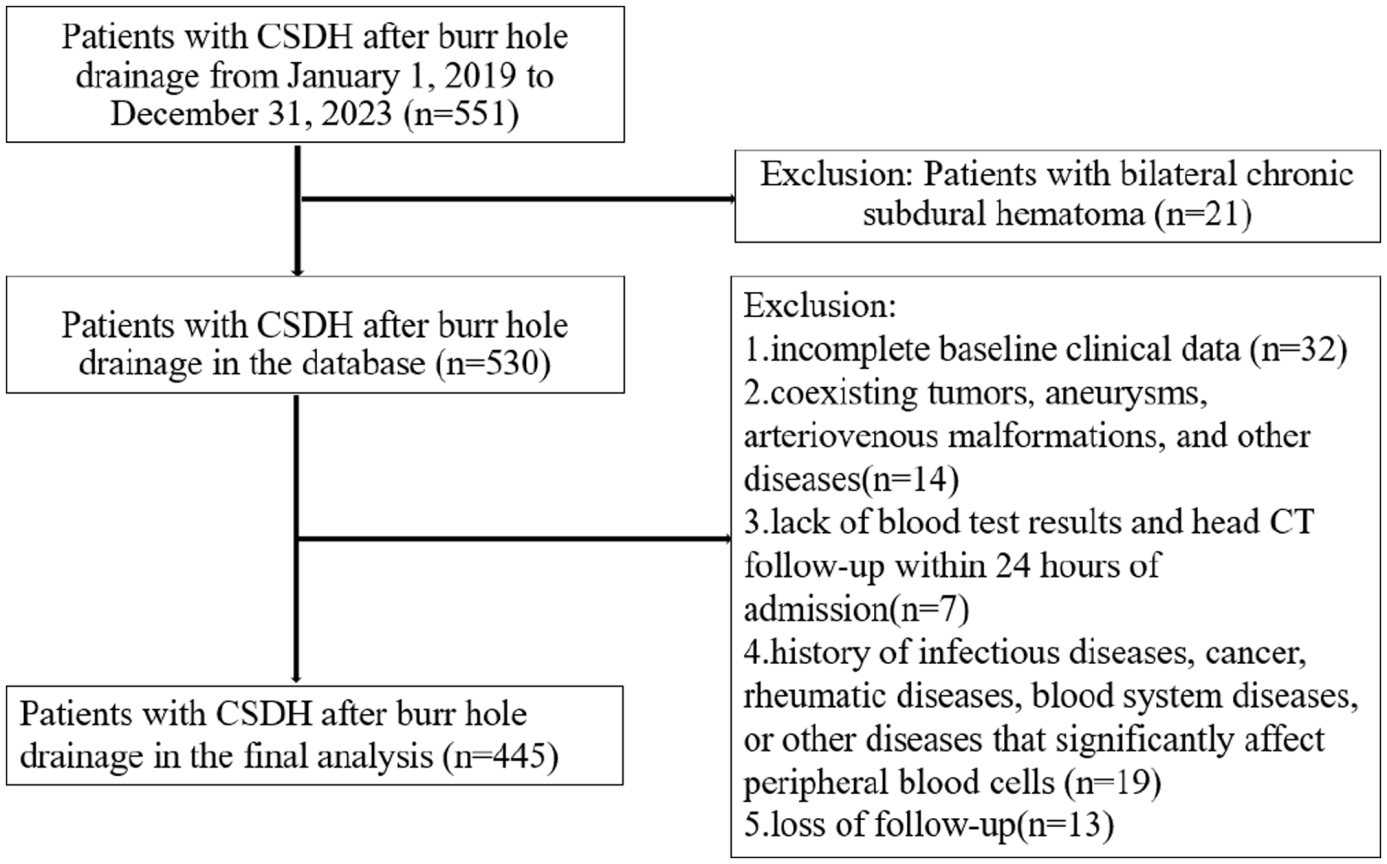
The flowchart of this study. CSDH, chronic subdural hematoma; CT, computed tomography.

A total of 551 CSDH patients underwent burr hole drainage at our medical center between January 1, 2019, and December 31, 2023. In this study, we excluded patients with bilateral chronic subdural hematoma (*n* = 21), incomplete baseline clinical data (*n* = 32), coexisting tumors, aneurysms, arteriovenous malformations, and other diseases (*n* = 14), lack of blood test results and CT follow-up within 24 h of admission (*n* = 7), history of infectious diseases, cancer, rheumatic diseases, blood system diseases, or other diseases that significantly affect peripheral blood cells (*n* = 19), and loss of follow-up (*n* = 13). Finally, 445 patients were enrolled in the final analysis (Figure 1).

The baseline characteristics and hematological biochemical markers of the enrolled patients are presented in Table 1. The median age was 67 (IQR 57–75) years, the median height was 164.0 (IQR 159.0–169.0) cm, the median weight was 63.0 (IQR 54.0–83.0) kg, the median BMI was 24.11 (IQR 20.15–30.10) kg/m^2^, and 72.81% were male. Among these patients, 90 (20.22%) developed the poor prognosis, 278 (62.47%) had hypertension, 44 (9.89%) had diabetes, 38 (8.54%) had ischemic stroke history, 72 (16.18%) had smoking history, 82 (18.43%) had alcohol abuse, and 32 (7.19%) had anticoagulation or antiplatelet therapy. The median thickness of hemorrhage was 1.00 (IQR 0.70–1.20) cm, and 137 (30.79%) patients had brain herniation. Among the 137 patients with brain herniation, imaging data revealed 108 cases of subfalcine herniation, 29 cases of uncal herniation, and no tonsillar herniation. The median admission GCS was 14.0 (IQR 12.0–15.0). Moreover, the median value of SIRI was 2.790 (IQR 1.530–5.378) 10^9^/L, the median value of SII was 652.993 (IQR 366.419–1294.851) 10^9^/L, and the median value of NLR was 3.876 (IQR 2.356–7.856) within 24 h of admission.

**Table 1:** Baseline characteristics of patients.

Variables	Total patients (*N* = 445)	Good prognosis (*N* = 355)	Poor prognosis (*N* = 90)	*p*-value
Age (years)	67.0(57.0–75.0)	68.0(57.0–76.0)	67.0(56.0–75.0)	0.502
Gender (%)				0.147
Male	324(72.81%)	264(74.37%)	60(66.67%)	
Female	121(27.19%)	91(25.63%)	30(33.33%)	
Height (cm)	164.0(159.0–169.0)	164.0(159.0–169.5)	163.0(158.3–168.0)	0.323
Weight (kg)	63.0(54.0–83.0)	65.0(54.0–83.0)	61.0(53.3–84.0)	0.582
BMI (kg/m^2^)	24.11(20.15–30.10)	24.32(20.07–30.47)	23.34(20.72–28.98)	0.998
Hypertension (%)				0.051
N	278(62.47%)	230(64.79%)	48(53.33%)	
Y	167(37.53%)	125(35.21%)	42(46.67%)	
Diabetes (%)				0.556
N	401(90.11%)	318(89.58%)	83(92.22%)	
Y	44(9.89%)	37(10.42%)	7(7.78%)	
Ischemic stroke history (%)				0.001**
N	407(91.46%)	333(93.80%)	74(82.22%)	
Y	38(8.54%)	22(6.20%)	16(17.78%)	
Anticoagulation or antiplatelet therapy (%)				0.002**
N	413(92.81%)	337(94.93%)	76(84.44%)	
Y	32(7.19%)	18(5.07%)	14(15.56%)	
Smoke (%)				0.108
N	373(83.82%)	303(85.35%)	70(77.78%)	
Y	72(16.18%)	52(14.65%)	20(22.22%)	
Alcohol (%)				0.127
N	363(81.57%)	295(83.10%)	68(75.56%)	
Y	82(18.43%)	60(16.90%)	22(24.44%)	
Brain herniation (%)				<0.001****
N	308(69.21%)	269(75.77%)	39(43.33%)	
Y	137(30.79%)	86(24.23%)	51(56.67%)	
Hematoma-thickness (cm)	1.00(0.70–1.20)	0.70(0.70–1.00)	1.20(0.90–1.20)	<0.001****
Admission GCS	14.0(12.0–15.0)	15.0(13.0–15.0)	9.5(6.0–13.0)	<0.001****
RBC (10^9^/L)	4.01(3.48–4.35)	4.03(3.49–4.33)	3.94(3.47–4.55)	0.691
HGB (10^9^/L)	119.0(104.0–131.0)	121.0(104.0–131.0)	118.0(103.0–127.8)	0.247
NEU (10^9^/L)	4.96(3.28–7.56)	4.41(3.00–6.70)	7.15(5.06–9.70)	<0.001****
MONO (10^9^/L)	0.70(0.49–0.92)	0.69(0.49–0.89)	0.75(0.53–1.14)	0.073
LYM (10^9^/L)	1.15(0.80–1.57)	1.21(0.88–1.62)	0.84(0.64–1.22)	<0.001****
PLT (10^9^/L)	167.0(125.0–213.0)	169.0(126.5–212.0)	165.0(116.0–226.0)	0.713
SIRI (10^9^/L)	2.79(1.53–5.38)	2.27(1.31–4.18)	6.12(3.28–11.05)	<0.001****
SII (10^9^/L)	652.99(366.42–1,294.85)	567.20(334.64–1,150.79)	1,275.92(695.98–2,182.76)	<0.001****
NLR	3.88(2.36–7.86)	3.38(2.18–6.09)	8.03(5.54–13.92)	<0.001****

Non-normally distributed numerical variables are represented as median and Interquartile range (IQR). BMI, body mass index; GCS, Glasgow Coma Scale; RBC, red blood cell; HGB, hemoglobin; NEU, neutrophil; MONO, monocyte; LYM, lymphocyte; PLT, platelet; SIRI, systemic inflammatory response Index; SII, systemic immune-inflammation index; NLR, neutrophil-to-lymphocyte ratio. The significance of bold data: *p* < 0.05.

### Risk factors for poor prognosis

At the 1-month follow-up after discharge, 90 (20.22%) patients achieved the poor prognosis. Compared with the good prognosis group, the SIRI, SII, and NLR were significantly higher in the poor prognosis group (all *p* < 0.001), as shown in Table 1. Univariate analysis (Table 1) revealed that ischemic stroke history (*p* = 0.001), brain herniation (*p* < 0.001), admission GCS (*p* < 0.001), anticoagulation or antiplatelet therapy (*p* = 0.002), hematoma thickness (*p* < 0.001), neutrophil (*p* < 0.001), and lymphocyte (*p* < 0.001) were significantly associated with poor prognosis.

### Multivariate logistic regression of factors related to poor prognosis

Moreover, according to multivariate analysis (Table 2), ischemic stroke history (*p* = 0.009; OR = 3.1674; 95% CI 1.313–7.499), brain herniation (*p* = 0.012; OR = 2.439; 95% CI 1.204–4.903), admission GCS (*p* < 0.001; OR = 0.749; 95% CI 0.675–0.828), anticoagulation or antiplatelet therapy (*p* = 0.018; OR = 3.236; 95% CI 1.187–8.450),

**Table 2:** Univariate logistic regression analysis of factors related to poor prognosis.

Variables	OR (95%CI)	*p*-value
Ischemic stroke history	3.167(1.313–7.499)	0.009**
Brain herniation	2.439(1.204–4.903)	0.012*
Admission GCS	0.749(0.675–0.828)	<0.001****
NEU (10^9^/L)	0.961(0.853–1.072)	0.497
LYM (10^9^/L)	0.940(0.676–1.315)	0.701
Hematoma-thickness (cm)	1.144(0.813–1.574)	0.42
SIRI (10^9^/L)	1.082(1.017–1.159)	0.019*
SII (10^9^/L)	1.000(0.999–1.000)	0.097
NLR	1.105(1.010–1.225)	0.045*
Anticoagulation or antiplatelet therapy	3.236(1.187–8.450)	0.018*

OR, odds ratio; CI, confidence interval; GCS, Glasgow Coma Scale; NEU, neutrophil; LYM, lymphocyte; SIRI, systemic inflammatory response Index; SII, systemic immune-inflammation index; NLR, neutrophil-to-lymphocyte ratio. The significance of bold data: *p* < 0.05.

SIRI (*p* = 0.019; OR = 1.082; 95% CI 1.017–1.159), and NLR (*p* = 0.045; OR = 1.105; 95% CI 1.010–1.225) were independent risk factors for poor prognosis. SII (*p* = 0.097) was not significant in multivariate regression analysis.

### Predictive ability and model-fitting of SIRI and NLR

**Figure 2:**
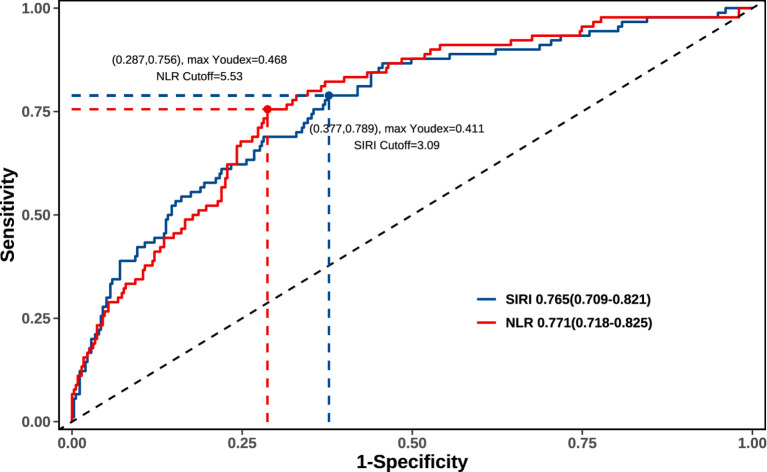
The receiver operating characteristic (ROC) curve of SIRI and NLR.

According to the Receiver Operating Characteristic (ROC) curve, the Area Under the Curve (AUC) value of SIRI was 0.765 (95% CI = 0.709–0.821), and the optimal cutoff point was 3.09 × 10^9^/L (sensitivity 78.9%, specificity 37.7%). The AUC value of NLR was 0.771 (95% CI = 0.718–0.825), and the optimal cutoff point was 5.53 (sensitivity 75.6%, specificity 28.7%) (Figure 2). Predictive models were conducted to further evaluate the predictive accuracy of the SIRI and NLR (Table 3). The basic model included 4 independent variables (history of ischemic stroke, brain herniation, admission GCS, anticoagulant or antiplatelet therapy) but exclude peripheral blood indicators. The “Basic model + NLR” had the C-index value (0.861) and AIC value (325.61). The “Basic model + SIRI” had the C-index value (0.862) and the lowest AIC (321.98), indicating the best model-fitting. The “Basic model + SIRI + NLR” had the AIC value (322.00) and the highest C-index value (0.865), indicating the best predictive accuracy (Table 3).

**Table 3:** Predictive models for predicting poor prognosis of CSDH patients after burr hole drainage.

Model	C-index (95%CI)	AIC
Basic model	0.842(0.797–0.887)	337.51
Basic model + SIRI	0.862(0.820–0.904)	321.98
Basic model + NLR	0.861(0.821–0.900)	325.61
Basic model + SIRI + NLR	0.865(0.824–0.905)	322.00

Basic model: history of ischemic stroke, cerebral hernia, admission GCS, anticoagulant or antiplatelet therapy. C-index, Harrell’s concordance index; AIC, Akaike information criterion; CI, confidence interval; SIRI, systemic inflammatory response Index; NLR, neutrophil-to-lymphocyte ratio.

### Nomogram for the “basic model + SIRI + NLR”

**Figure 3:**
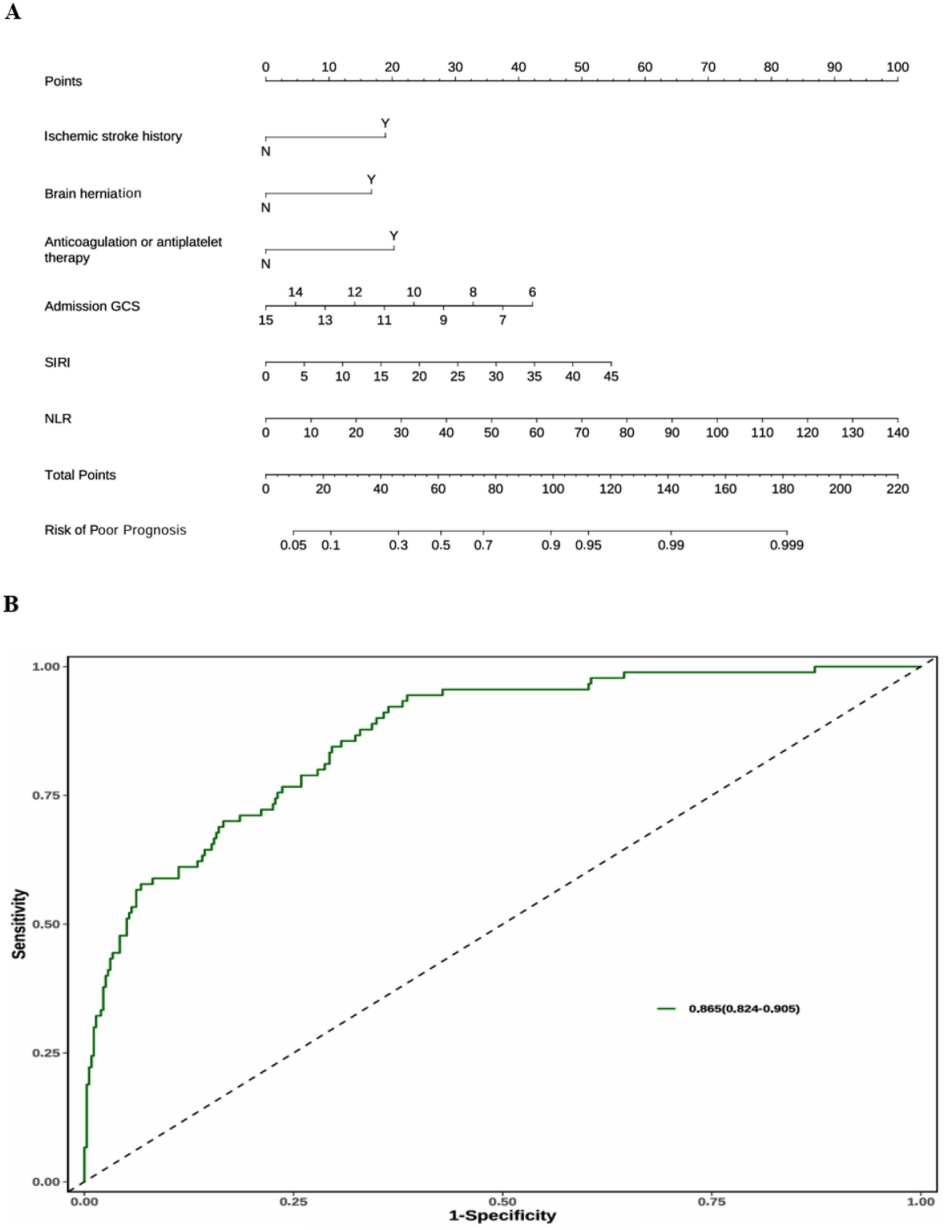
**(A)** The nomogram of the “Basic model + SIRI + NLR.” **(B)** The receiver operating characteristic (ROC) curve of the nomogram.

The “Basic model + SIRI + NLR” has the highest prediction accuracy (C-index = 0.865), and the AIC value difference between the “Basic model + SIRI” (the best model-fitting) is 0.02 (AIC 322.00 vs. 321.98). Therefore, we used the “Basic model + SIRI + NLR” to create a nomogram that explains the utility of logistic regression and clearly displays the effect of SIRI and NLR on prognosis (Figure 3A). A total score was calculated with the ischemic stroke history, brain herniation, admission GCS, anticoagulation or antiplatelet therapy, SIRI, and NLR. On the point scale axis, a score was assigned to each of these variables’ value. A total score could be easily calculated by summing each individual score, and by projecting the total score to the lower total point scale, we could estimate the probability of the poor prognosis. According to the Receiver Operating Characteristic (ROC) curve of the Nomogram (Figure 3B), the Area Under the Curve (AUC) value was 0.865 (95% CI 0.824–0.905).

## Discussion

CSDH has always been characterized as a mild, benign, and easily treatable condition, most likely due to its surgical simplicity (13, 41, 42). However, the incidence of CSDH has almost tripled, and the number of surgeries has doubled during the past two decades due to aging (2, 6). By 2030, it is expected that there will be 60,000 cases per year in the United States (7). CSDH is associated with increased mortality and a poorer prognosis than previously thought (13, 41, 43–45). Minor craniocerebral trauma and bridge vein injury do not fully explain the etiology of CSDH; instead, inflammation and the immune system are thought to play an important role in its development (13, 17–20, 46, 47). SIRI, SII, and NLR are novel systemic inflammatory and immune markers derived from peripheral blood that can more accurately reflect the balance of immune and inflammatory responses (28, 29, 31). SIRI, SII, and NLR play essential prognostic roles in a variety of diseases (32–37). However, few studies have reported the prognostic value of SIRI, SII, and NLR in CSDH patients following burr hole draining.

To our knowledge, this is the first report on the predictive relevance of SIRI in functional outcomes of CSDH patients after burr hole drainage. Our findings show that SIRI, NLR, ischemic stroke history, brain herniation, admission GCS, and anticoagulation or antiplatelet therapy are independent risk factors for poor prognosis of postoperative functional outcomes in CSHD patients (Table 2). When combined with non-hematologic predictors, SIRI performs better than NLR in terms of model-fitting and prediction accuracy (Table 3). However, SII had no significant prognostic value in our study (Table 2). Perhaps this is because platelet levels at admission better reflect the patient’s coagulation capability during the acute phase, but there is no significant link with chronic inflammatory levels. Patients undergoing surgery should have platelet levels within the normal range to guarantee the safety of the treatment. Our findings are consistent with several previous research on the prognosis of patients with CSDH (13, 15, 16, 47–52). Unsurprisingly, admission GCS was significantly associated with the poor prognosis (13, 15, 16, 51). Churl-Su et al. discovered that admission GCS < 13 was significantly related with a poor prognosis of CSDH (15). Furthermore, a 12-month follow-up research indicated that admission GCS has a substantial impact on CSDH outcomes (51). As previously reported, anticoagulation or antiplatelet therapy can independently predict the prognosis of CSDH patients (48–50, 52–54), and our study confirmed this. According to earlier researches (52, 53, 55, 56), CSDH patients who experienced an ischemic stroke had considerably adverse postoperative functional outcomes. CSDH with brain herniation usually progresses rapidly and has a poor prognosis, suggesting emergency burr holes or craniotomy for hematoma drainage (57–62).

This study investigated the prognostic value of SIRI and NLR because their components are so similar. NLR has been widely used as a valid indication for a variety of disorders, and previous research has shown it to be a strong predictor of prognosis in CSDH patients (28, 29, 31, 63). Kayalar et al. discovered that CSDH patients had a significantly increased risk of poor prognosis when NLR exceeded 2.8, with a 1-unit rise in the NLR raising the risk by 5.2 times (64). According to a Brazilian study, increased postoperative NLR is an independent risk factor for poor CSDH outcomes (63). SIRI’s calculating formula is neutrophil count × monocyte count/lymphocyte count, which is equivalent to monocyte count × NLR. As previously stated, inflammation plays a significant role in the pathophysiology of CSDH (17–20). The local inflammatory response of CSDH is mediated by a variety of inflammatory and immunological cells, including neutrophils, lymphocytes, and monocytes (17, 20, 23, 24). Numerous cytokines, including chemokines, are released by these inflammatory cells after they deposit in the CSDH membrane, aggravating the tendency of inflammatory and immunological cells to congregate. The biological activity of these chemokines can promote the production of new blood vessels in the CSDH envelope, prolong local inflammation, and increase hematoma formation, all of which contribute to a poor prognosis for CSDH. Nonetheless, other chemokines enhance CSDH repair by reducing local inflammation and inhibiting capsule neovascularization (18). Thus, the balance of local inflammation in CSDH is represented in the balance of inflammatory cells and inflammatory chemicals, which is critical for the prognosis (18, 20, 25). The ratio of inflammatory cells in peripheral blood can be used to reflect local inflammation and predict a patient’s prognosis because it is linked to the body’s systemic inflammatory response (26, 27). Therefore, peripheral blood inflammation markers such as SIRI and NLR might partially reflect the balance of CSDH local inflammation and have prognostic value for CSDH patients following burr hole draining. We recognize that dynamic changes in inflammatory markers may be a better predictor of outcome. Due to the lack of unified blood checks after the surgery, we were not able to obtain these postoperative inflammatory markers.

Neutrophils are the primary components of SIRI and NLR. Neutrophils are critical for the inflammatory response because they are the first immune system cells to respond to infection and tissue injury (65). According to a study on cerebral hemorrhage, neutrophils, the first white blood cells to infiltrate the brain from peripheral blood, remain elevated for a week (37). Vaibhav et al. discovered that patients with recurrent CSDH may have elevated neutrophil counts, indicating ongoing inflammation in the subdural region (48). However, a prolonged inflammatory response wears out the immune system, resulting in immuno-depression syndrome, which reduces systemic immunity activity, suppresses systemic cellular immunological responses, and causes a decrease in peripheral blood lymphocyte subsets (29, 66). This immune-suppression phenomenon is known as stroke-induced immune-depression syndrome (SIDS) (66). Persistent local CSDH inflammation can also produce immunological suppression, which reduces peripheral blood lymphocytes, impairs the body’s ability to resist, increases susceptibility to infection, and leads to a poor prognosis. This is comparable to the phenomenon of immune suppression caused by a stroke. Our study revealed that neutrophils (*p* < 0.001) and lymphocytes (*p* < 0.001) were associated with a poor prognosis in univariate analysis. However, they did not appear to be independent risk factors in multivariate analysis. As a result, the NLR (neutrophil-to-lymphocytes ratio) can better reflect the balance of inflammation and predict the prognosis of CSDH when neither neutrophils nor lymphocytes can independently reflect the balance of local inflammation.

Monocytes are the distinguishing factor between SIRI and NLR. Monocytes, a kind of white blood cells, can develop into a variety of tissue macrophages and dendritic cells (67). Monocytes are mononuclear myeloid cells that originate in the bone marrow and circulate in the bloodstream (68). Studies of intracerebral hemorrhage have indicated that a high monocyte count at admission is an independent predictor of hematoma growth and a poor prognosis (36). In our analysis, monocytes (*p* = 0.073) did not predict a poor prognosis. One of the primary features of inflammation is the migration of monocytes to wounded tissues and the circulation (69). During inflammation, monocytes circulate in the bloodstream and extravasate into inflamed tissue, triggering a general leukocyte recruitment cascade that includes rolling, adhesion, and migration. Monocytes’ ability to mobilize and transport themselves to where they are needed is critical to their role in strengthening immune defense during infection (67). These recruited monocytes contribute to the spread of inflammation. Monocytes play an important role in immune defense and tissue healing, but they also cause tissue necrosis during inflammation. Given the importance of monocytes in inflammatory reactions, this could partly explain why the SIRI (a combination of monocytes and NLR) has prognostic value. When combined with the basic model (history of ischemic stroke, brain herniation, admission GCS, anticoagulant or antiplatelet therapy), the SIRI has better predictive accuracy (C-index 0.862 vs. 0.861) and model-fitting (AIC 321.98 vs. 325.61) than NLR.

Finally, neutrophils, monocytes, and lymphocytes play an important role in CSDH’s inflammatory response. The use of the inflammatory index (SIRI or NLR) can more accurately reflect the complexity of the inflammatory response because the single inflammatory cell measure is sensitive to variables such as race, dehydration, and specimen preparation (37). Furthermore, SIRI and NLR rely on indicators obtained by a regular blood test, which is a hematological test required for nearly all CSDH patients. Its price and convenience of usage reduce the financial burden on healthcare and encourage widespread adoption.

Currently, “middle meningeal artery embolization” is regarded as a new adjunctive treatment method. Many studies have found that it improves the prognosis of CSDH. Unfortunately, none received the middle meningeal artery embolization among the patients we included. We believe that as clinical research progresses, it will be beneficial to incorporate it into the prediction model. There are several limitations in this study. First, this was a retrospective study conducted in a single center. Second, we used short-term outcome endpoints since our center does normal follow-up for surgical patients at 1 month post-discharge. The long-term functional prognosis may vary. In forthcoming studies, we will validate these indicators and investigate their efficacy in predicting illness recurrence. Third, some occult infections cannot be discovered early using clinical and laboratory criteria, thus leading to bias. To validate our findings, more large-sample multicenter prospective studies may be required. We need to track patients with long-term prognosis and recurrence in order to better grasp the predictive ability of crucial variables.

## Conclusion

To our knowledge, this is the first study to investigate the prognostic value of admission SIRI in CSDH patients. In this study, SIRI and NLR had prognostic value in CSDH patients after burr hole drainage, while SII had no prognostic significance. When combined with the basic model, the SIRI has better predictive accuracy and model-fitting than NLR.

## Data Availability

The datasets presented in this study can be found in online repositories. The names of the repository/repositories and accession number(s) can be found in the article/Supplementary material.
